# P-1518. Activity of Ceftolozane/Tazobactam, Imipenem/Relebactam and Comparators Against Clinical MDR and DTR non-*Morganellaceae* Enterobacterales and *Pseudomonas aeruginosa* Stratified by Patient Age and Hospital Ward: SMART United States 2020-2022

**DOI:** 10.1093/ofid/ofae631.1687

**Published:** 2025-01-29

**Authors:** Mark G Wise, C Andrew DeRyke, John Esterly, Karri A A Bauer, Fakhar Siddiqui, Katherine Young, Mary Motyl, Daniel F Sahm

**Affiliations:** IHMA, Schaumburg, Illinois; IHMA, Schaumburg, Illinois; Merck & Co., Inc., Rahway, New Jersey; Merck & Co, Inc, Kenilworth, New Jersey; Merck & Co., Inc., Rahway, New Jersey; Merck, Rahway, New Jersey; Merck, Rahway, New Jersey; IHMA, Schaumburg, Illinois

## Abstract

**Background:**

Hospital ward and patient age are factors that can guide in the selection of empiric therapy. Imipenem/relebactam (IMR) is a combination of imipenem with the β-lactamase inhibitor relebactam, an inhibitor of class A and C β-lactamases. Ceftolozane/tazobactam (C/T) combines ceftolozane, an anti-pseudomonal cephalosporin, with tazobactam. We evaluated the activity of IMR, C/T and comparators against isolates of non-Morganellaceae Enterobacterales (NME) and *Pseudomonas aeruginosa* that were collected in the United States as part of the SMART surveillance program from 2020 to 2022 with data stratified by hospital ward (ICU versus general wards), and by patient age (< 60 and ≥60 years old).

**Figure:**
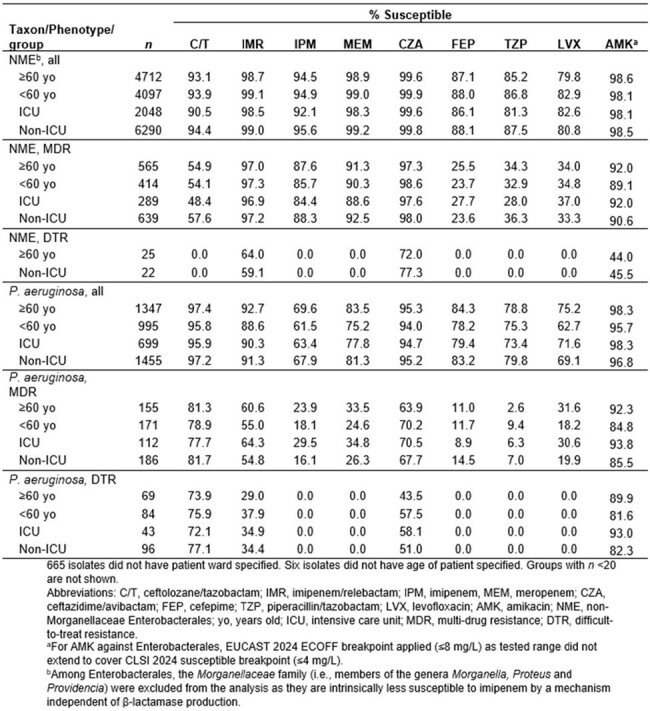

**Methods:**

In 2020-2022, 26 clinical labs in the US each collected up to 250 consecutive Gram-negative pathogens per year. MICs were determined using CLSI broth microdilution and interpreted with 2024 CLSI breakpoints. Multi-drug resistance (MDR) was defined as resistance to ≥3 sentinel agents (amikacin, aztreonam, cefepime, ceftazidime [Enterobacterales only], colistin, imipenem, levofloxacin, and piperacillin/tazobactam). Difficult-to-treat resistance (DTR) was defined as non-susceptibility to all β-lactams (including aztreonam, ceftazidime, cefepime, imipenem, meropenem, piperacillin-tazobactam), and fluoroquinolones (levofloxacin).

**Results:**

Susceptibility to commonly used β-lactams was lower among isolates from ICU patients compared to those in non-ICU wards, but no clear pattern was linked to patient age. Against the NME, IMR was among the most active agents inhibiting >98% of the isolates in all strata (Table). Meropenem, ceftazidime/avibactam and amikacin also inhibited >98% of the isolates. IMR retained activity against the MDR NME, as >96.9% of the isolates were susceptible. Against *P. aeruginosa*, C/T was the first or second (after amikacin) most active agent versus isolates in all strata, with >95% susceptible. C/T remained active against ≥77% of MDR and ≥72% of DTR isolates, 7-30 percentage points higher than ceftazidime/avibactam.

**Conclusion:**

IMR is an important treatment option for patients with infections caused by NME, while C/T is an excellent choice against *P. aeruginosa,* regardless of patient age or treatment in the ICU.

**Disclosures:**

**Daniel F. Sahm, PhD**, Pfizer, Inc.: Advisor/Consultant

